# Global monthly sectoral water use for 2010–2100 at 0.5° resolution across alternative futures

**DOI:** 10.1038/s41597-023-02086-2

**Published:** 2023-04-11

**Authors:** Zarrar Khan, Isaac Thompson, Chris R. Vernon, Neal T. Graham, Thomas B. Wild, Min Chen

**Affiliations:** 1 Joint Global Change Research Institute, Pacific Northwest National Laboratory, 5825 University Research Ct., Suite 3500, College Park, MD 20740 USA; 2 Department of Forest and Wildlife Ecology, College of Agriculture & Life Sciences, University of Wisconsin – Madison, Russell Labs, 1630 Linden Drive, Madison, WI 53706 USA

**Keywords:** Hydrology, Environmental sciences

## Abstract

Water usage is closely linked with societal goals that are both local and global in scale, such as sustainable development and economic growth. It is therefore of value, particularly for long-term planning, to understand how future sectoral water usage could evolve on a global scale at fine resolution. Additionally, future water usage could be strongly shaped by global forces, such as socioeconomic and climate change, and the multi-sector dynamic interactions those forces create. We generate a novel global gridded monthly sectoral water withdrawal and consumption dataset at 0.5° resolution for 2010–2100 for a diverse range of 75 scenarios. The scenarios are harmonized with the five Shared Socioeconomic Pathways (SSPs) and four Representative Concentration Pathways (RCPs) scenarios to support its usage in studies evaluating the implications of uncertain human and earth system change for future global and regional dynamics. To generate the data, we couple the Global Change Analysis Model (GCAM) with a land use spatial downscaling model (Demeter), a global hydrologic framework (Xanthos), and a water withdrawal downscaling model (Tethys).

## Background & Summary

This paper documents a global monthly gridded (0.5° resolution) sectoral water withdrawal and consumption dataset that contains conditional projections of water usage (from 2010 to 2100) across a range of future socio-economic and climate scenarios. This dataset is important because it quantifies the sources of demand-side pressures on scarce water resources globally under diverse future scenarios. Mekonnen & Hoekstra 2016^1^ (also cited in the UN World Water Development Report 2022^2^) estimated that roughly 71% (4.1 billion people) of the world’s population was exposed to water scarcity at least one month in the year over the period from 1996 to 2005. In their more recent study, Van Vliet *et al*. 2021^3^ estimate global water scarcity over the period from 2000 to 2010 to range from 30% (without water quality considered) to 40% (when also including water quality). Global water scarcity is expected to increase across the globe with critical implications for sustainable development^4–8^. Recent studies highlight that future water scarcity is primarily driven by human water demands rather than climate impacts on water availability^4,9^. Additionally, irrigation water demands have been shown to have the largest relative impact on water scarcity^5,6,10^. Furthermore, water access, availability and demands are highly localized, with large energy and economic costs associated with water transfers, and thus a regional understanding of water use is essential^11,12^. This paper accounts for all of these key factors by providing a transparent and open-source dataset and accompanying methodology that captures the key drivers of future water scarcity (water use for human activities) at a fine spatio-temporal scale (0.5° resolution and monthly) and with added detail on irrigation water use by crop types.

Past studies^13–15^ that have evaluated global gridded water use at monthly resolution have been limited to historical analyses. Other studies, such as World Resources Institute (WRI) 2019^16^, look at future water withdrawals but only at an annual time resolution and up to 2040 with sectoral detail divided into domestic, industry, agriculture and livestock sectors. In this paper we offer a finer spatiotemporal resolution for future projections compared to previous studies applied to a broader suite of socioeconomic and climate forcing scenarios. Additionally, we provide more detail in the irrigation sector which includes 13 different crop types by coupling our water demand model with a land allocation model. Table 1 compares the key features in this study to a representative set of previous studies that have analysed global water use. Table 1 highlights that, compared to previous studies, our study captures additional sectoral detail (especially by irrigated crop types) and a more diverse set of future scenarios.

**Table 1:** Comparison of selected global water use studies.

	Water Use Types	Sectors	Additional Sectors	Spatial Scope	Temporal Scope	Scenarios
Khan *et al*. 2022 (This study)	- Withdrawals - Consumption	- Mining - Domestic - Electricity - Livestock - Industry - Irrigation	(13 Crops) Biomass, Corn, Fiber Crop, Misc Crop, Oil Crop, Other Grain, Palm Fruit, Rice, Root Tuber, Sugar Crop, Wheat, Fodder Herb, and Fodder Grass	- Global - 0.5deg gridded	**Historical** - 2010 - Monthly **Future/Simulated** - 2015 to 2100 - Monthly	**Historical** 2010 **Future** - SSPs 1 to 5 - RCP2.6, 4.5, 6.0, 8.5 - 5 CMIP5 GCMs (GFDL, HADGEM, IPSL, MIROC, NORESM)
Aqueduct (WRI) (2019, 2015)^16,38^	- Withdrawals - Consumption	- Domestic - Industry - Agriculture - Livestock	—	- Global - 0.083deg (historical) - 0.5deg (future)	**Historical** - 1990–2014 - Monthly **Future/Simulated:** - 2020, 2030, 2040 - Annual	**Historical** PCR-GLOBWB 2 Outputs **Future** - SSP2, SSP3 - RCP4.5, RCP8.5 - 6 CMIP5 GCMs (CCSM4, CNRM-CM5, GFDL-ESM2M, INMCM4, MPI-ESM-LR, MRI-CGCM3)
Huang *et al*.^13^	- Withdrawals - Consumption	- Mining - Domestic - Electricity - Livestock - Industry - Irrigation	—	- Global - 0.5deg gridded	**Historical** - 1971–2010 - Monthly	**Historical** 4 GHMs: WaterGAP, H08, LPJml, PCR-GLOBWB)
Wada *et al*.^14^	- Withdrawals - Consumption	- Domestic - Livestock - Industry - Irrigation	- Paddy - Non-paddy	- Global - 0.5deg gridded	**Historical** - 1979 - 2010 - Daily	**Historical** - 1979–2010
Hanasaki *et al*.^5^	- Withdrawals	- Municipal - Industry - Irrigation	—	- Global - 0.5deg gridded	**Historical** - 2000 to 2100 - Daily	**Historical** 2000 **Future** - SSPs 1 -5 - RCP2.6, 4.5, 6.0, 8.5
Mekonnen & Hoekstra 2011^15^	- Consumption (blue water footprint)	- Total	- Additional datasets available for crops, industrial products and livestock^39–41^	- Global - 0.5deg gridded	**Historical** - 1996 - 2005 - Monthly	**Historical** Outputs of water balance model

This study thus addresses the critical need for future projections of distributed water demand at a fine resolution so that scientists and water managers can start to explore and plan for future water needs. The dataset could also directly support the growing MultiSector dynamics research literature, particularly scenario-based studies of the future interactions between water and other sectors (e.g., energy and land) across scales in a global context^17–19^. The diverse set of 75 scenarios we produce supports scenario-based water demand uncertainty analysis by varying key elements of human and earth system change. The entire dataset can be downloaded from a dataverse online repository^20^ (10.7910/DVN/VIQEAB) and is accompanied by a meta-repository (https://jgcri.github.io/khan-etal_2022_tethysSSPRCP/) that provides detailed figures and workflows for interested readers.

**Fig. 1:**
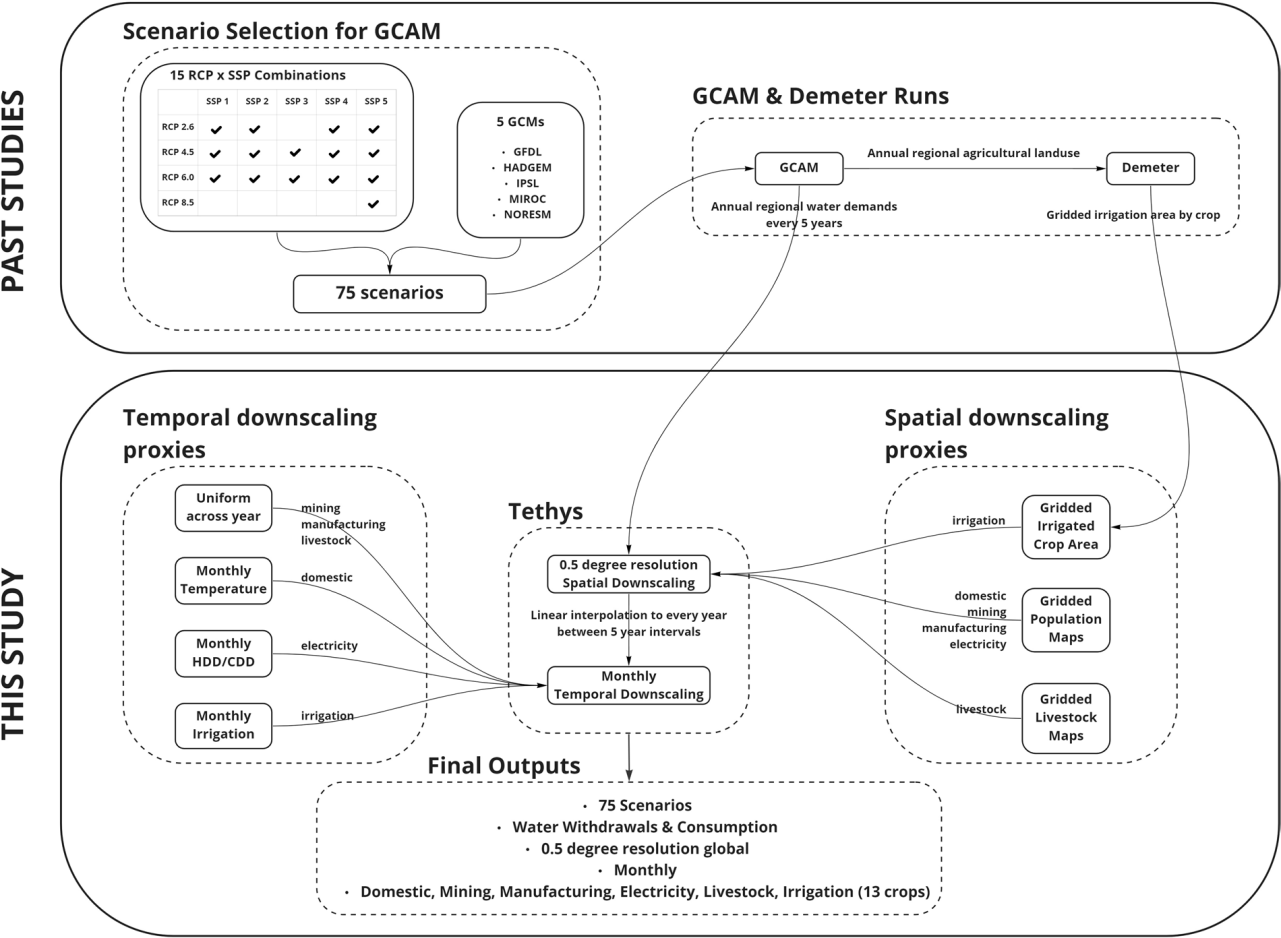
Study workflow showing the 75 scenarios are a combination of 4 Representative Concentration Pathways (RCPs), 5 Shared Socioeconomic Pathways (SSPs) and 5 Global Climate Models (GCMs). 15 viable combinations of SSPs and RCPs were combined with each of the 5 GCMs to arrive at the final 75 scenarios which are were then used to generate the corresponding GCAM scenarios which were then passed onto Demeter. Annual water demands from the GCAM runs (Graham *et al*. 2020^4^) and irrigated crop land area from the Demeter study (Chen *et al*. 2020^27^) were then passed onto Tethys to generate the final results of this study.

We generated this dataset by linking together multiple models and datasets designed to explore the dynamic interactions among energy, water, and land systems at global scale and gridded resolution. Central to our modeling workflow is the Global Change Analysis Model (GCAM^4^), an integrated tool for exploring the coarse regional dynamics of the coupled human-Earth system and the response of this system to global change, including human system and climate system changes into the future. Tethys^21^ then spatially and temporally downscales outputs from GCAM to grid resolution. We enhance Tethys’ projections of irrigation water usage by coupling it with Demeter^22^, a high-resolution downscaling model that uses GCAM outputs to calculate global gridded land-use change. With the combination of GCAM and Demeter, Tethys is able to project water withdrawal and consumption demands for 6 sectors (domestic, electricity generation, irrigation, livestock, industry and mining). The irrigation sector is further divided into 13 different crop types (biomass, corn, fiber crop, miscellaneous crops, oil crop, other grain, palm fruit, rice, root tuber, sugar crop, wheat, fodder herb, and fodder grass). Withdrawal refers to the total volume of water that is extracted by a user from a water source. While some of this withdrawn water may be returned to its original source (e.g., a river), a remaining portion (referred to as consumption) may not returned to the system (e.g., evaporated water). To capture a range of futures reflecting diverse global change across the human and Earth systems, we used 75 scenarios comprised of a combination of 4 Representative Concentration Pathways (RCPs)^23^, 5 Shared Socioeconomic Pathways (SSPs)^24^, and 5 Global Climate Models (GCMs) from the Inter-sectoral Impact Model Intercomparison Project (ISIMIP)^25^ protocol 2b. 15 viable combinations of the SSPs and RCPs were combined with each of the 5 GCMs to arrive at the final 75 scenarios. Graham *et al*. 2020^4^ provides the details on these original GCAM runs for the 75 scenarios which included a characterization of demand-side narratives corresponding to the SSPs for the water sector^26^. The GCAM outputs were then passed on to the Demeter model to produce the downscaled irrigated crop land area for 13 different crops in the study by Chen *et al*. 2020^27^. The combined outputs from the GCAM study and the Demeter study were used in this study to calculate the final downscaled water demand results. The entire workflow of data from the original scenarios through GCAM and Demeter to Tethys is shown in Fig. 1.

## Methods

**Fig. 2:**
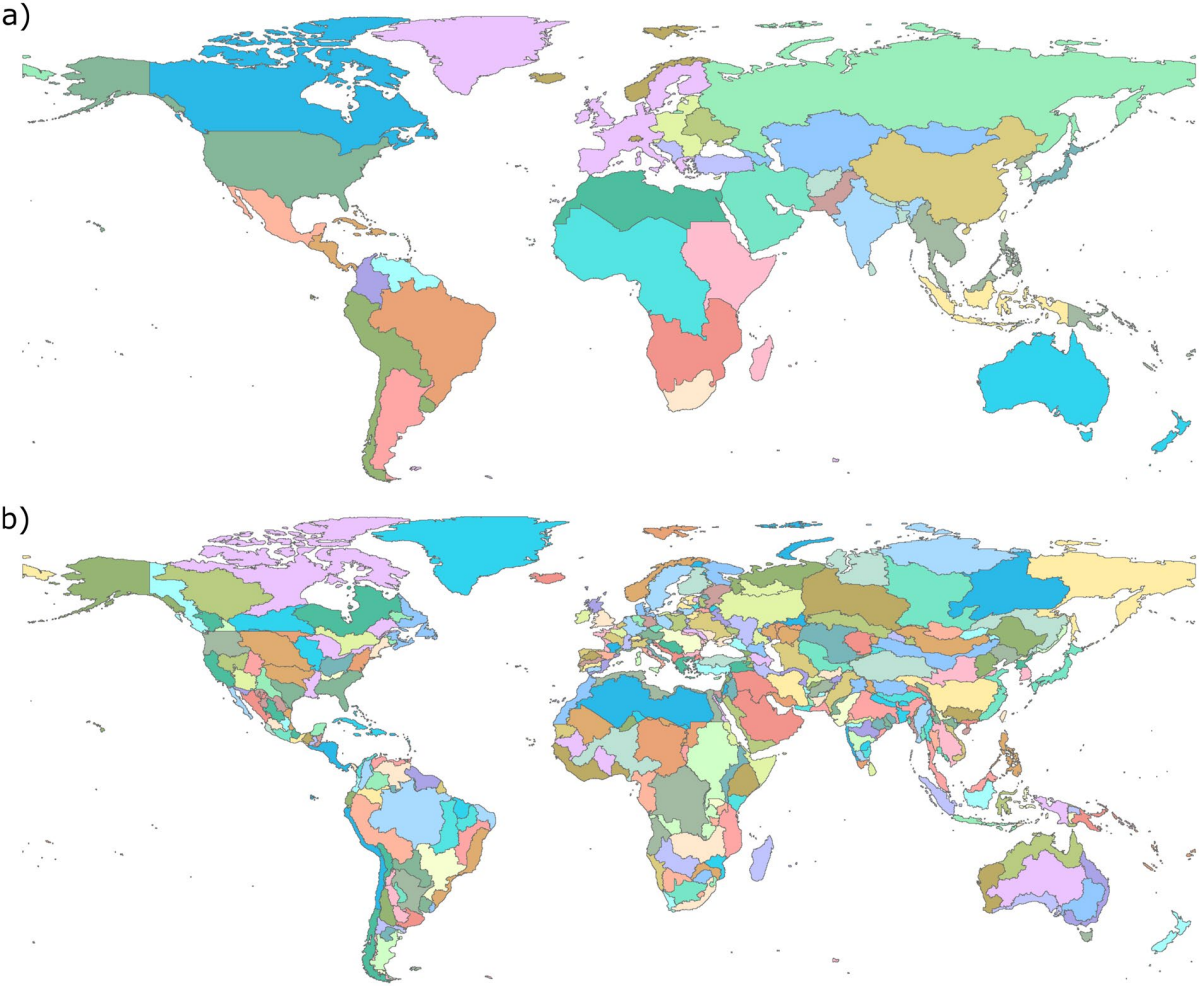
Water withdrawals and consumption from GCAM by a) 32 GCAM regions for domestic, mining, power generation, industry, and livestock sectors and b) 434 GCAM region and basin intersections for the irrigation sector.

**Fig. 3:**
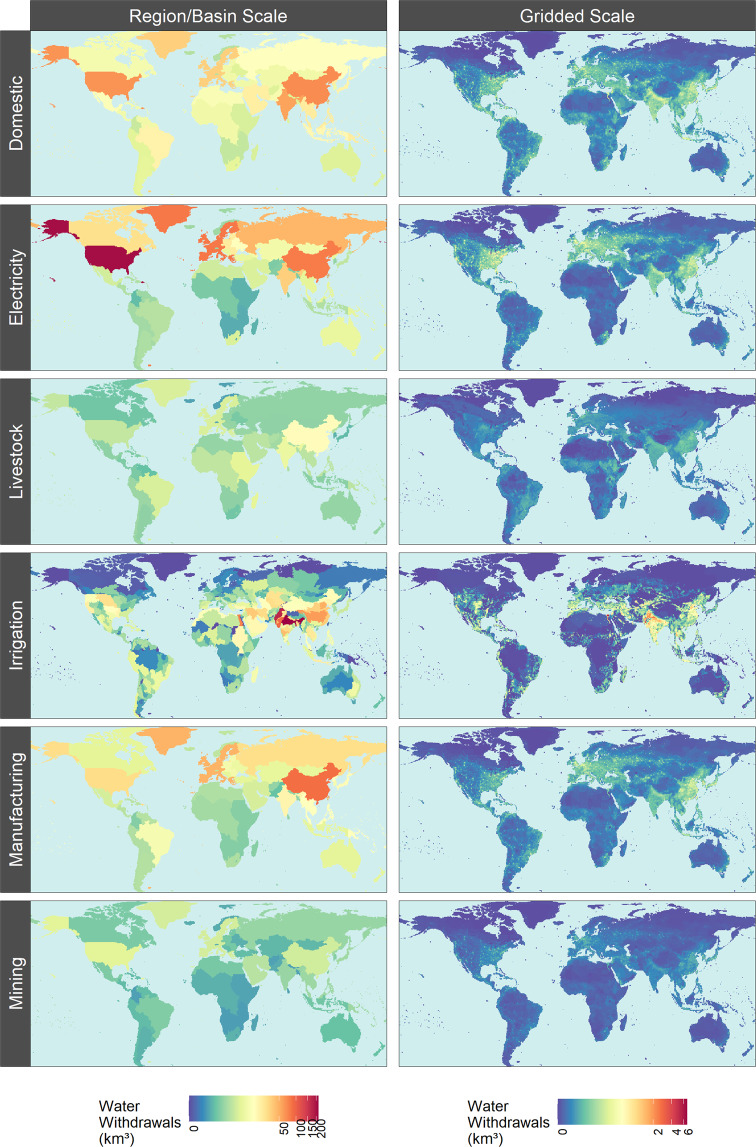
Example outputs of Tethys spatial downscaling of 2010 water withdrawals by sector from GCAM regions and basins to 0.5° × 0.5° grid cells.

GCAM produces water withdrawal and consumption outputs for 32 regions for the domestic, mining, power generation, industry, and livestock sectors and for 434 region-basin intersections for the irrigation sector as shown in Fig. 2. (These spatial boundaries^28^ are determined by Moirai^29^, the land data system used by GCAM). Tethys v1.3.1^30^ was used to downscale the water withdrawals and consumption outputs from GCAM onto a 0.5° by 0.5° grid as shown in Fig. 3. Of the 259,200 possible grid cells at this resolution (360 × 720), only the 67,420 cells categorized as land are considered. The Tethys outputs focus only on demand-side dynamics, so they make no distinctions regarding the water supply sources used to meet the demands (i.e., surface water, groundwater, desalinated water), though GCAM does make this distinction. While many adjacent regions differ largely in total water demand, most of this demand is directly related to total population or land area, and often concentrated in a few cells, such as those containing cities. As a result, spatial distributions at the border are smoother than they appear on the region scale map, without additional consideration of the boundaries by Tethys.

### Spatial downscaling – non-agriculture

Spatial downscaling for non-agricultural (domestic, electricity, manufacturing, and mining), water withdrawals and consumption in each grid cell are assumed to be proportional to that cell’s population as compared to the larger GCAM region within which that grid cell is located. The population data set used for this paper is from “Gridded Population of the World” (SEDAC, 2016)^31^. Tethys uses the nearest available year, which for this paper was 2010 in 2010, and 2015 in all other years. Each region’s population is determined by taking the sum of population over all cells belonging to that region. For each of these sectors, Tethys calculates the water withdrawals and consumption as shown in Eq. 1, 2 for a given cell by:

1 $${{\rm{withdrawal}}}_{{\rm{cell}}}={{\rm{withdrawal}}}_{{\rm{region}}}\times \frac{{{\rm{population}}}_{{\rm{cell}}}}{{{\rm{population}}}_{{\rm{region}}}}$$

2 $${{\rm{consumption}}}_{{\rm{cell}}}={{\rm{consumption}}}_{{\rm{region}}}\times \frac{{{\rm{population}}}_{{\rm{cell}}}}{{{\rm{population}}}_{{\rm{region}}}}$$

Large groups of cells with the same value are a by-product of the areal-weighting method used in the proxy, where coarse census data are evenly distributed.

### Spatial downscaling – livestock

Spatial downscaling of livestock water use is calculated using gridded global maps from the FAO gridded livestock of the world (Wint and Robinson, 2007)^32^ dataset for six types of livestock (cattle, buffalo, sheep, goats, pigs, and poultry). GCAM outputs are organized into five types (beef, dairy, pork, poultry, and “sheepgoat”) and these are first reorganized to match the six types from Wint and Robinson, 2007^32^ using ratios for each region estimated from the dataset. The ratios are stored in two files that are used as inputs to Tethys: bfracFAO2005.csv (“buffalo fraction”) and gfracFAO2005.csv (“goat fraction”). The following formulas are used to map the water withdrawals and consumption values for the five GCAM livestock types to the six livestock types from Wint and Robinson, 2007^32^ for each region:

3 $${\rm{buffalo}}=\left({\rm{beef}}+{\rm{dairy}}\right)\times {\rm{buffalo}}\_{\rm{fraction}}$$

4 $${\rm{cattle}}=\left({\rm{beef}}+{\rm{dairy}}\right)\times \left(1-{\rm{buffalo}}\_{\rm{fraction}}\right)$$

5 $${\rm{goat}}=\left({\rm{sheepgoat}}\right)\times {\rm{goat}}\_{\rm{fraction}}$$

6 $${\rm{sheep}}=\left({\rm{sheepgoat}}\right)\times (1-{\rm{goat}}\_{\rm{fraction}})$$

No adjustment is required for pork (pigs) or poultry. After this, downscaling for each livestock type is very similar to downscaling the nonagricultural sectors, with the exception that the respective livestock population (heads) is used as the proxy instead of human population.

7 $${{\rm{withdrawal}}}_{{\rm{animal}},{\rm{cell}}}={{\rm{withdrawal}}}_{{\rm{animal}},{\rm{region}}}\times \frac{{{\rm{heads}}}_{{\rm{animal}},{\rm{cell}}}}{{{\rm{heads}}}_{{\rm{animal}},{\rm{region}}}}$$

8 $${{\rm{consumption}}}_{{\rm{animal}},{\rm{cell}}}={{\rm{consumption}}}_{{\rm{animal}},{\rm{region}}}\times \frac{{{\rm{heads}}}_{{\rm{animal}},{\rm{cell}}}}{{{\rm{heads}}}_{{\rm{animal}},{\rm{region}}}}$$

The results for each of the six types are then added together to get the total livestock withdrawal and consumption for each cell:

9 $${{\rm{withdrawal}}}_{{}_{{\rm{livestock}},{\rm{cell}}}}=\left(\begin{array}{c}{{\rm{withdrawal}}}_{{\rm{cattle}},{\rm{cell}}}+\\ \begin{array}{c}{{\rm{withdrawal}}}_{{\rm{buffalo}},{\rm{cell}}}+\\ {{\rm{withdrawal}}}_{{\rm{sheep}},{\rm{cell}}}+\\ {{\rm{withdrawal}}}_{{\rm{goat}},{\rm{cell}}}+\\ {{\rm{withdrawal}}}_{{\rm{pigs}},{\rm{cell}}}+\\ {{\rm{withdrawal}}}_{{\rm{poultry}},{\rm{cell}}}\end{array}\end{array}\right)$$

10 $${{\rm{consumption}}}_{{\rm{livestock}},{\rm{cell}}}=\left(\begin{array}{c}{{\rm{consumption}}}_{{\rm{cattle}},{\rm{cell}}}+\\ \begin{array}{c}{{\rm{consumption}}}_{{\rm{buffalo}},{\rm{cell}}}+\\ {{\rm{consumption}}}_{{\rm{sheep}},{\rm{cell}}}+\\ {{\rm{consumption}}}_{{\rm{goat}},{\rm{cell}}}+\\ {{\rm{consumption}}}_{{\rm{pigs}},{\rm{cell}}}+\\ {{\rm{consumption}}}_{{\rm{poultry}},{\rm{cell}}}\end{array}\end{array}\right)$$

### Spatial downscaling – irrigation

GCAM irrigation water withdrawal and consumption outputs are organized by 13 crop types: Biomass, Corn, Fiber Crop, Miscellaneous Crop, Oil Crop, Other Grain, Palm Fruit, Rice, Root Tuber, Sugar Crop, Wheat, Fodder Herb, and Fodder Grass. By downscaling GCAM output, Demeter^22^ provides a spatial landcover breakdown for each crop type. Because the Demeter outputs used in this study were harmonized to match the land areas of a base map, they are first converted back to be consistent with GCAM. Using these adjusted irrigation area values for each crop, cell withdrawal and consumption values are given by:

11 $${{\rm{withdrawal}}}_{{\rm{crop}},{\rm{cell}}}={{\rm{withdrawal}}}_{{\rm{crop}},{\rm{region}},{\rm{basin}}}\times \frac{{{\rm{area}}}_{{\rm{crop}},{\rm{cell}}}}{{{\rm{area}}}_{{\rm{crop}},{\rm{region}},{\rm{basin}}}}$$

12 $${{\rm{consumption}}}_{{\rm{crop}},{\rm{cell}}}={{\rm{consumption}}}_{{\rm{crop}},{\rm{region}},{\rm{basin}}}\times \frac{{{\rm{area}}}_{{\rm{crop}},{\rm{cell}}}}{{{\rm{area}}}_{{\rm{crop}},{\rm{region}},{\rm{basin}}}}$$

In cases where the GCAM outputs for a region-basin have nonzero irrigation of a crop type, but Demeter shows no corresponding cells (due to the harmonization with the base map), the distribution is assumed to be proportional to land area. Note that in the current version of Tethys (v.1.3.1) used in this paper, biomass is also downscaled uniformly within a region-basin intersection (with respect to land area), as given by:

13 $${{\rm{withdrawal}}}_{{\rm{biomass}},{\rm{cell}}}={{\rm{withdrawal}}}_{{\rm{biomass}},{\rm{region}}}\times \frac{{{\rm{area}}}_{{\rm{cell}}}}{{{\rm{area}}}_{{\rm{region}},{\rm{basin}}}}$$

14 $${{\rm{consumption}}}_{{\rm{biomass}},{\rm{cell}}}={{\rm{consumption}}}_{{\rm{biomass}},{\rm{region}}}\times \frac{{{\rm{area}}}_{{\rm{cell}}}}{{{\rm{area}}}_{{\rm{region}},{\rm{basin}}}}$$

The total irrigation sector value for a cell is the sum of that cell’s values for all 13 crops.

#### Temporal downscaling – domestic

Temporally downscaling domestic withdrawal and consumption uses the following formula from Wada *et al*., 2011^33^. The R parameter described below is from Huang *et al*. 2018^13^ and temperature data is from Weedon *et al*. 2014^34^. Withdrawals and consumption for each month of a year for each cell are given by the formula:

15 $${{\rm{withdrawal}}}_{{\rm{month}}}=\frac{{{\rm{withdrawal}}}_{{\rm{year}}}}{12}\left[\left(\frac{{{\rm{temp}}}_{{\rm{month}}}-{{\rm{temp}}}_{{\rm{mean}}}}{{{\rm{temp}}}_{{\rm{\max }}}-{{\rm{temp}}}_{{\rm{\min }}}}\right){\rm{R}}+1\right]$$

16 $${{\rm{consumption}}}_{{\rm{month}}}=\frac{{{\rm{consumption}}}_{{\rm{year}}}}{12}\left[\left(\frac{{{\rm{temp}}}_{{\rm{month}}}-{{\rm{temp}}}_{{\rm{mean}}}}{{{\rm{temp}}}_{{\rm{\max }}}-{{\rm{temp}}}_{{\rm{\min }}}}\right){\rm{R}}+1\right]$$

Where:

temp_month_ = Average temperature for the month

temp_mean_ = Mean monthly temperature for the year

temp_max_ = Max monthly temperature for the year

temp_min_ = Min monthly temperature for the year

R = Parameter representing the relative difference of water use between the warmest and coolest months of the year

#### Temporal downscaling – electricity generation

Water withdrawal and consumption for electricity generation each month are assumed to be proportional to the amount of electricity consumed, using the formula developed in Voisin *et al*., 2013^35^:

17 $${{\rm{withdrawal}}}_{{\rm{month}}}={{\rm{withdrawal}}}_{{\rm{year}}}\left[{{\rm{\rho }}}_{{\rm{b}}}\left(\begin{array}{c}{{\rm{\rho }}}_{{\rm{h}}}\frac{{{\rm{HDD}}}_{{\rm{month}}}}{{{\rm{HDD}}}_{{\rm{year}}}}+\\ {{\rm{\rho }}}_{{\rm{c}}}\frac{{{\rm{CDD}}}_{{\rm{month}}}}{{{\rm{CDD}}}_{{\rm{year}}}}+\\ {{\rm{\rho }}}_{{\rm{u}}}\frac{1}{12}\end{array}\right)+{{\rm{\rho }}}_{{\rm{it}}}\frac{1}{12}\right]$$

18 $${{\rm{consumption}}}_{{\rm{month}}}={{\rm{consumption}}}_{{\rm{year}}}\left[{{\rm{\rho }}}_{{\rm{b}}}\left(\begin{array}{c}{{\rm{\rho }}}_{{\rm{h}}}\frac{{{\rm{HDD}}}_{{\rm{month}}}}{{{\rm{HDD}}}_{{\rm{year}}}}+\\ {{\rm{\rho }}}_{{\rm{c}}}\frac{{{\rm{CDD}}}_{{\rm{month}}}}{{{\rm{CDD}}}_{{\rm{year}}}}+\\ {{\rm{\rho }}}_{{\rm{u}}}\frac{1}{12}\end{array}\right)+{{\rm{\rho }}}_{{\rm{it}}}\frac{1}{12}\right]$$

Where:

ρ_b_ = Proportion of electricity used for buildings

ρ_it_ = Proportion of electricity used for industry and transportation

ρ_b_+ρ_it_ = 1

ρ_h_ = Proportion of electricity used for buildings heating

ρ_c_ = Proportion of electricity used for buildings cooling

ρ_u_ = Proportion of electricity used for buildings other

ρ_h_+ρ_c_+ρ_u_ = 1

HDD = Heating Degree Days

CDD = Cooling Degree Days

Heating degree days (HDD) and cooling degree days (CDD) are indicators for the amount of electricity used to heat and cool buildings, and are calculated from mean daily outdoor air temperature. HDD for a month is the sum of (18 °C -temperature_day_) across all days where temperature is less than 18 degrees Celsius. CDD is the sum of (temperature_day_ – 18°C) across all days where temperature is greater than 18°C. Annual HDD and CDD are the sum of their respective monthly values.

Tethys uses HDD, CDD, and ρ values for each cell from the nearest available year in the input files listed at the end of this subsection, which is 2010 for this data set.

The formula is modified for cells with low annual HDD or CDD as described in Huang *et al*., 2018^13^, since these may not have heating or cooling services despite nonzero values of ρ_h_ or ρ_c_.

When HDD_year_<650, the HDD term is removed (leaving only CDD) and ρ_h_ is reallocated to the cooling proportion, giving:

19 $${{\rm{withdrawal}}}_{{\rm{month}}}={{\rm{withdrawal}}}_{{\rm{year}}}\left[{{\rm{\rho }}}_{{\rm{b}}}\left(\begin{array}{c}\left({{\rm{\rho }}}_{{\rm{h}}}+{{\rm{\rho }}}_{{\rm{c}}}\right)\frac{{{\rm{CDD}}}_{{\rm{month}}}}{{{\rm{CDD}}}_{{\rm{year}}}}+\\ {{\rm{\rho }}}_{{\rm{u}}}\frac{1}{12}\end{array}\right)+{{\rm{\rho }}}_{{\rm{it}}}\frac{1}{12}\right]$$

20 $${{\rm{consumption}}}_{{\rm{month}}}={{\rm{consumption}}}_{{\rm{year}}}\left[{{\rm{\rho }}}_{{\rm{b}}}\left(\begin{array}{c}\left({{\rm{\rho }}}_{{\rm{h}}}+{{\rm{\rho }}}_{{\rm{c}}}\right)\frac{{{\rm{CDD}}}_{{\rm{month}}}}{{{\rm{CDD}}}_{{\rm{year}}}}+\\ {{\rm{\rho }}}_{{\rm{u}}}\frac{1}{12}\end{array}\right)+{{\rm{\rho }}}_{{\rm{it}}}\frac{1}{12}\right]$$

When CDD_year_<450, the CDD term is removed (leaving only HDD) and ρ_c_ is reallocated to the cooling proportion, giving:

21 $${{\rm{withdrawal}}}_{{\rm{month}}}={{\rm{withdrawal}}}_{{\rm{year}}}\left[{{\rm{\rho }}}_{{\rm{b}}}\left(\begin{array}{c}\left({{\rm{\rho }}}_{{\rm{h}}}+{{\rm{\rho }}}_{{\rm{c}}}\right)\frac{{{\rm{HDD}}}_{{\rm{month}}}}{{{\rm{HDD}}}_{{\rm{year}}}}+\\ {{\rm{\rho }}}_{{\rm{u}}}\frac{1}{12}\end{array}\right)+{{\rm{\rho }}}_{{\rm{it}}}\frac{1}{12}\right]$$

22 $${{\rm{consumption}}}_{{\rm{month}}}={{\rm{consumption}}}_{{\rm{year}}}\left[{{\rm{\rho }}}_{{\rm{b}}}\left(\begin{array}{c}\left({{\rm{\rho }}}_{{\rm{h}}}+{{\rm{\rho }}}_{{\rm{c}}}\right)\frac{{{\rm{HDD}}}_{{\rm{month}}}}{{{\rm{HDD}}}_{{\rm{year}}}}+\\ {{\rm{\rho }}}_{{\rm{u}}}\frac{1}{12}\end{array}\right)+{{\rm{\rho }}}_{{\rm{it}}}\frac{1}{12}\right]$$

When annual HDD and CDD are both below their respective thresholds (<650 for HDD and <450 for CDD), all sources of monthly variation vanish and the formula reduces to

23 $${{\rm{withdrawal}}}_{{\rm{month}}}=\frac{{{\rm{withdrawal}}}_{{\rm{year}}}}{12}$$

24 $${{\rm{consumption}}}_{{\rm{month}}}=\frac{{{\rm{consumption}}}_{{\rm{year}}}}{12}$$

#### Temporal downscaling – livestock, manufacturing and mining

For livestock, manufacturing, and mining, a uniform distribution is applied. The withdrawal or consumption for the year is divided between months according to the number of days.

25 $${{\rm{withdrawal}}}_{{\rm{month}}}={{\rm{withdrawal}}}_{{\rm{year}}}\times \frac{{{\rm{days}}}_{{\rm{month}}}}{{{\rm{days}}}_{{\rm{year}}}}$$

26 $${{\rm{consumption}}}_{{\rm{month}}}={{\rm{consumption}}}_{{\rm{year}}}\times \frac{{{\rm{days}}}_{{\rm{month}}}}{{{\rm{days}}}_{{\rm{year}}}}$$

#### Temporal Downscaling – Irrigation

Temporal downscaling for irrigation water withdrawal and consumption is based on weighted irrigation profiles for each of the 235 basins. Gridded monthly irrigation withdrawal values from the PCR-GLOBWB global hydrological (from Huang *et al*. 2018^13^, original data from ISIMIP^36^) model are averaged across the years 1971–2010, then aggregated to the basin scale. The monthly irrigation withdrawal percentages for a basin are applied to all crops in each of its cells.

27 $${{\rm{withdrawal}}}_{{\rm{month}}}={{\rm{withdrawal}}}_{{\rm{year}}}\times {{\rm{percent}}}_{{\rm{basin}},{\rm{month}}}$$

28 $${{\rm{consumption}}}_{{\rm{month}}}={{\rm{consumption}}}_{{\rm{year}}}\times {{\rm{percent}}}_{{\rm{basin}},{\rm{month}}}$$

In the event that the model has no monthly data for a basin with nonzero irrigation, the profile of the nearest available basin is used.

## Data Records

Data outputs from this experiment have been minted and are available in the repository indicated in Table 2. A meta-repository with detailed information on the workflows to produce the data is also available and shown in Table 2.

**Table 2:** Data records.

Record	Details	Location
Output Dataset^20^	Data outputs from experiment	10.7910/DVN/VIQEAB
Supporting Meta-repository	Meta-repository with detailed workflows for experiment	https://jgcri.github.io/khan-etal_2022_tethysSSPRCP/index.html

The dataset contains separate files with names which start with a combination of the following SSP, RCP, GCM and water usage type:

- **SSP:** ssp1, ssp2, spp3, spp4, spp5
- **RCP:** rcp26, rcp45, rcp60, rcp85
- **GCM:** gfdl, hadgem, ipsl, miroc, noresm
- **Water use type:** consumption, withdrawals

**Example 1:** ssp1_rcp26_gfdl_consumption_XXX

**Example 2:** ssp1_rcp26_gfdl_withdrawal_XXX

The datasets files have been then divided into sub-sets to manage their size. The following list shows the file structure for one of the SSP, RCP, GCM combinations:

- ssp1_rcp26_gfdl_consumption_crops_annual.zip
- ssp1_rcp26_gfdl_consumption_crops_monthly_1.zip
- ssp1_rcp26_gfdl_consumption_crops_monthly_2.zip
- ssp1_rcp26_gfdl_consumption_sectors_annual.zip
- ssp1_rcp26_gfdl_consumption_sectors_monthly_1.zip
- ssp1_rcp26_gfdl_consumption_sectors_monthly_2.zip

The files with “_crops_” in their names include data for individual crops while the files with “_sectors_” in their name include data for other aggregated sectors. The following expanded list shows the individual files inside the zipped files for the example ssp1_rcp26_gfdl cases. **“cd” stands for “consumption downscaled” and “tcd” stands for “temporal consumption downscaled”**:

- ssp1_rcp26_gfdl_consumption_crops_annual.zip
  - crops_cdirr_biomass_km3peryr.csv
  - crops_cdirr_Corn_km3peryr.csv
  - crops_cdirr_FiberCrop_km3peryr.csv
  - crops_cdirr_FodderGrass_km3peryr.csv
  - crops_cdirr_FodderHerb_km3peryr.csv
  - crops_cdirr_MiscCrop_km3peryr.csv
  - crops_cdirr_OilCrop_km3peryr.csv
  - crops_cdirr_OtherGrain_km3peryr.csv
  - crops_cdirr_PalmFruit_km3peryr.csv
  - crops_cdirr_Rice_km3peryr.csv
  - crops_cdirr_Root_Tuber_km3peryr.csv
  - crops_cdirr_SugarCrop_km3peryr.csv
  - crops_cdirr_Wheat_km3peryr.csv
- ssp1_rcp26_gfdl_consumption_crops_monthly_1.zip
  - crops_tcdirr_biomass_km3peryr.csv
  - crops_tcdirr_Corn_km3peryr.csv
  - crops_tcdirr_FiberCrop_km3peryr.csv
  - crops_tcdirr_FodderGrass_km3peryr.csv
  - crops_tcdirr_FodderHerb_km3peryr.csv
  - crops_tcdirr_MiscCrop_km3peryr.csv
  - crops_tcdirr_OilCrop_km3peryr.csv
- ssp1_rcp26_gfdl_consumption_crops_monthly_2.zip
  - crops_tcdirr_OtherGrain_km3peryr.csv
  - crops_tcdirr_PalmFruit_km3peryr.csv
  - crops_tcdirr_Rice_km3peryr.csv
  - crops_tcdirr_Root_Tuber_km3peryr.csv
  - crops_tcdirr_SugarCrop_km3peryr.csv
  - crops_tcdirr_Wheat_km3peryr.csv
- ssp1_rcp26_gfdl_consumption_sectors_annual.zip
  - cddom_km3peryr.csv(Domestic)
  - cdelec_km3peryr.csv(Electricity Generation)
  - cdirr_km3peryr.csv(Irrigation)
  - cdliv_km3peryr.csv(Livestock)
  - cdmfg_km3peryr.csv(Industry & manufacturing)
  - cdmin_km3peryr.csv(Mining)
  - cdnonag_km3peryr.csv(Aggregated non-agriculture)
  - cdtotal_km3peryr.csv(Total)
- ssp1_rcp26_gfdl_consumption_sectors_monthly_1.zip
  - tcddom_km3peryr.csv(Domestic)
  - tcdelec_km3peryr.csv(Electricity Generation)
  - tcdirr_km3peryr.csv(Irrigation)
- ssp1_rcp26_gfdl_consumption_sectors_monthly_2.zip
  - tcdliv_km3peryr.csv(Livestock)
  - tcdmfg_km3peryr.csv(Industry & manufacturing)
  - tcdmin_km3peryr.csv(Mining)

## Technical Validation

**Fig. 4:**
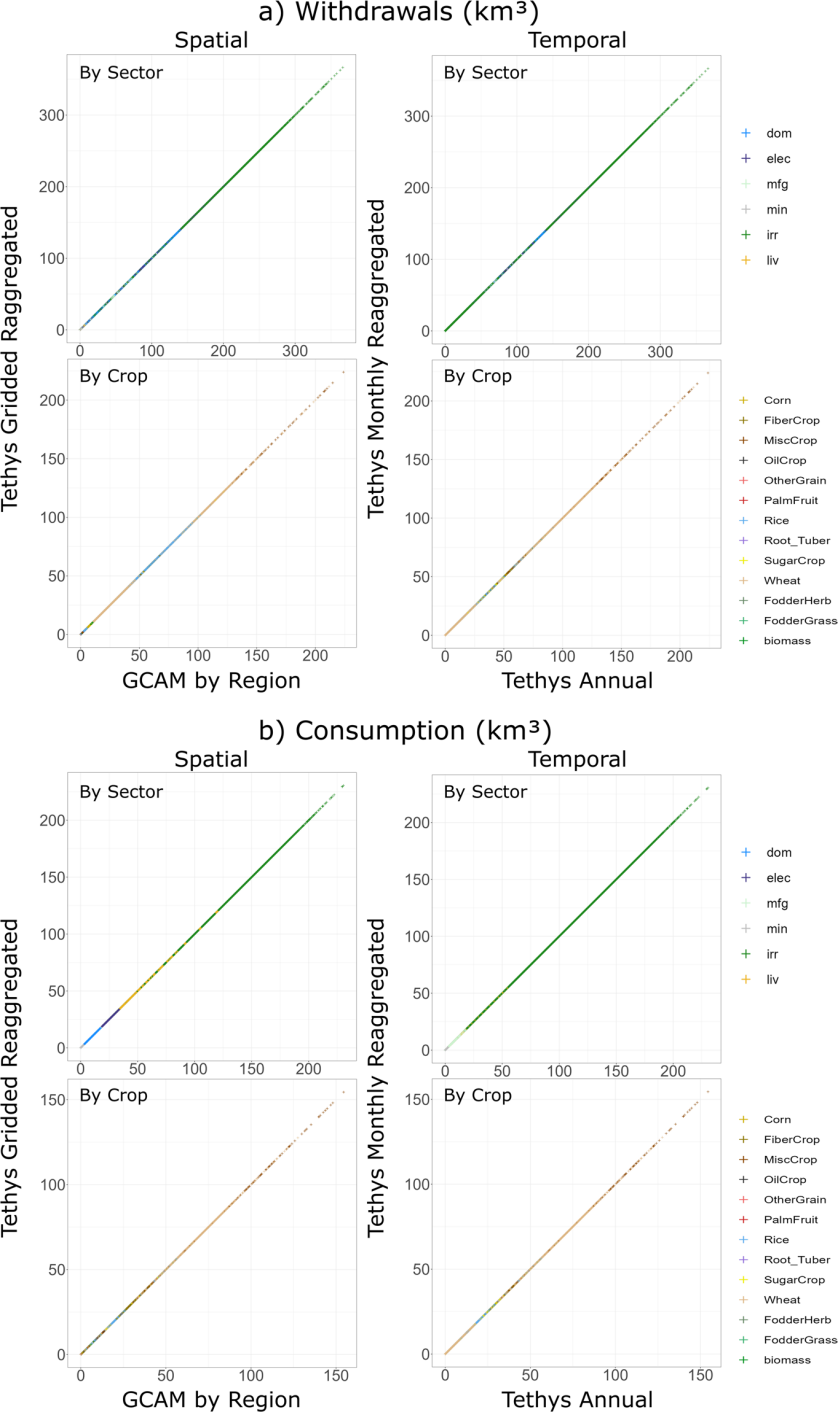
Validation of downscaled spatial and temporal Tethys water use. a) Water Withdrawals (km^3^) and b) Water Consumption (km^3^).

GCAM outputs are calibrated at a regional scale to match observed data for base year values as described in Graham *et al*. 2020^4^. Sectoral comparison between GCAM’s future water demand projections and other studies is carried out in the supporting information of Graham *et al*. 2018^26^. In this study, validation is limited to ensuring that the downscaling algorithms in Tethys are free of errors and there is no loss in values as a result of the temporal or spatial downscaling methodology. The results of this study were validated by re-aggregating spatial and temporal downscaled model outputs and comparing them to the original aggregated inputs. Figure 4a shows how the disaggregated water withdrawal values in km^3^ equal the original values both spatially for GCAM regions and temporally for annual values across sectors and crops. Figure 4b shows the same validation for how the disaggregated water consumption values in km^3^ equal the original values both spatially for GCAM regions and temporally for annual values across sectors and crops.

**Fig. 5:**
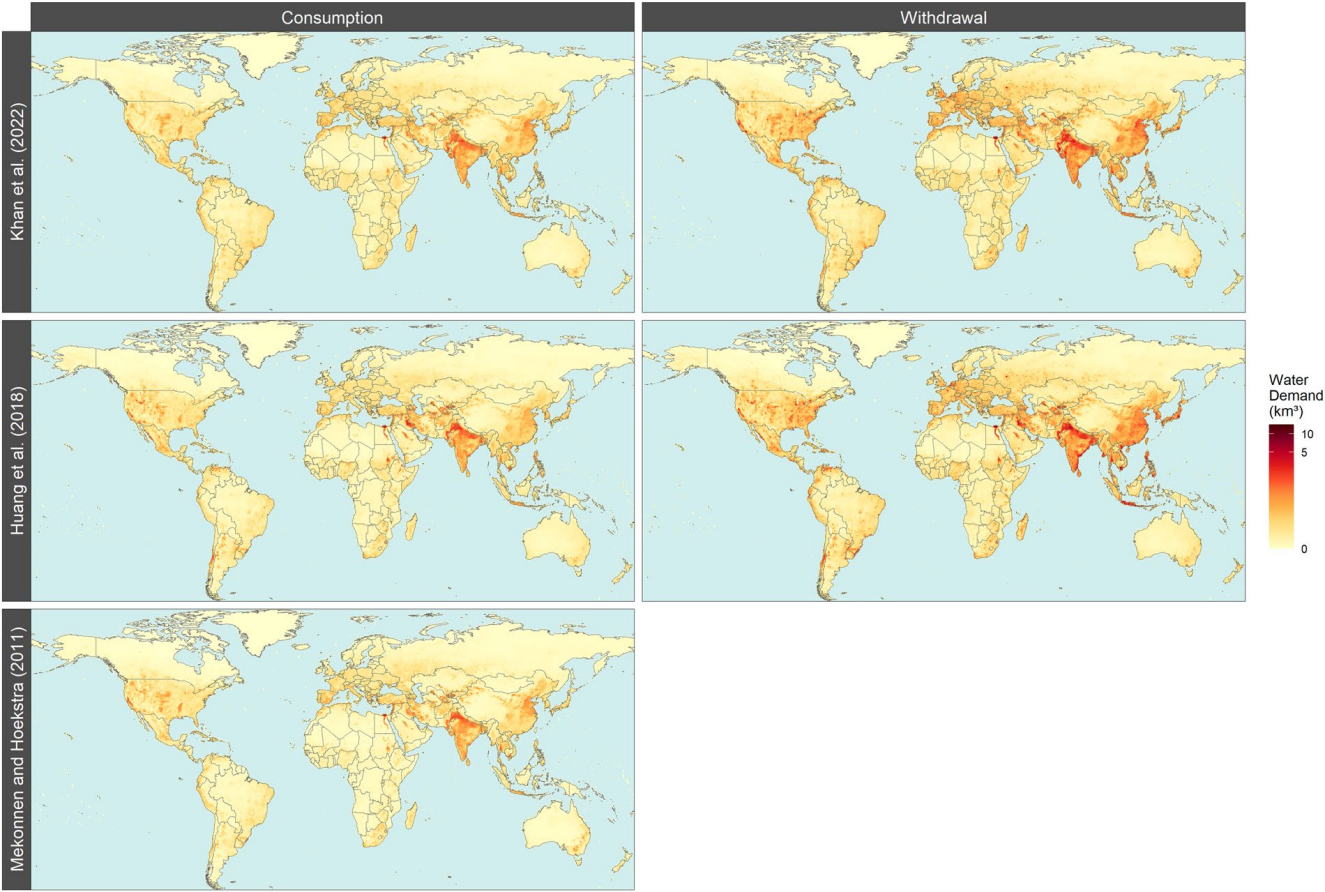
Spatial distribution of water withdrawals and consumption across this study (year 2010), Huang *et al*. 2018^13^ (year 2010) and Mekonnen, M.M. and Hoekstra, A.Y. 2011^15^ (average of years 1996–2005).

Additionally, Tethys outputs were also compared to results from two other studies: Huang *et al*. 2018^13^ and Mekonnen, M.M. and Hoekstra, A.Y. 2011^15^ as shown in Fig. 5. Given the larger number of variables and assumptions for future scenarios considered here, we limit the validation with other studies to historical data. Since this work is primarily concerned with the downscaling of existing projections to a gridded monthly scale, we look at how spatial and temporal patterns in the year 2010 (for which all scenarios are identical) compare to those of the chosen datasets.

Huang *et al*. 2018^13^, uses an earlier version of Tethys on historical data from 1971–2010. The underlying data have more regions and different totals, but many of the downscaling methods are identical, leading to similar results. For the non-agricultural sectors (domestic, electricity, manufacturing, and mining), the same underlying population map is used to downscale water use. For irrigation, Huang *et al*. 2018^13^ use United States Geological Survey (USGS) and Food and Agricultural Organization (FAO) AQUASTAT irrigation data, whereas the current version of Tethys uses crop landcover maps from Demeter. Consumption and withdrawals generally showed similar spatial patterns, with differences in assumptions regarding each region’s and sector’s consumption-to-withdrawal ratios accounting for some differences. There are also some differences in accounting. For example, in this study hydropower is included in the consumption for electricity generation category, which by itself is several times greater than the entire water consumption for electricity generation in Huang *et al*. 2018^13^.

The second data set we compared with is from Mekonnen, M.M. and Hoekstra, A.Y. 2011^15^. It contains monthly total blue water consumption values representing an average of years 1996–2005, which we compare to the base year values from 2010 from this study. The sectoral breakdown is different between the two datasets, but the datasets are at the same spatial-temporal resolution, so we compare monthly totals for each grid cell. Comparing datasets cell by cell is highly sensitive to local differences, and since our spatial downscaling is based on proxy quantities we do not expect every detail to be recreated exactly.

**Fig. 6:**
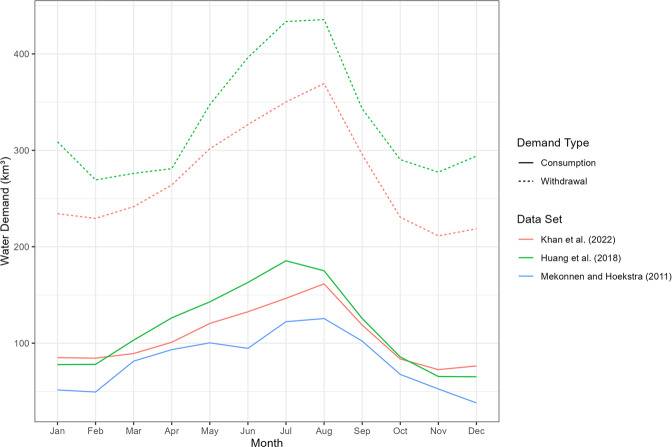
Temporal distribution of global water withdrawals and consumption across this study (year 2010), Huang *et al*. 2018^13^ (year 2010) and Mekonnen, M.M. and Hoekstra, A.Y. 2011^15^ (average of years 1996–2005).

Nonetheless, there is general agreement in the sub-regional patterns across the data sets as seen in Fig. 5. Figure 6 also shows similar sub-annual patterns across the dataset with some differences in total values being attributed to underlying data and year of the study.

**Table 3:** Model and data code availability.

Type	Details	Model Version	Data DOI	Model DOI
**Tethys**	Used to generate the data presented in this paper	v1.3.1	10.7910/DVN/VIQEAB^20^	10.5281/zenodo.6399488^30^
GCAM*	Water use data used as inputs for Tethys	v4.3.chen	10.7910/DVN/DYV29J^42^	10.5281/zenodo.3713432^43^
**Demeter**	Landuse change data used as input for Tethys	v1.chen	https://data.pnnl.gov/dataset/13192^44^	10.5281/zenodo.3713378^45^

* Note: For users wanting to explore the water consumption and withdrawal data directly from the original GCAM databases we provide a short R script at: https://github.com/JGCRI/khan-etal_2022_tethysSSPRCP/blob/v1-pre-publish/scripts/extract_water_data.R

(10.5281/zenodo.7636762).

## Usage Notes

Users are encouraged to explore the accompanying meta-repository (https://jgcri.github.io/khan-etal_2022_tethysSSPRCP/index.html), which provides detailed visualization across the various scenarios, sectors and time periods. Users can then download specific datasets for water withdrawal or consumption for relevant sectors, crops and desired SSP, RCP or GCM from the accompanying dataset repository^20^ (10.7910/DVN/VIQEAB) to analyze the raw data. Some example figures from the meta-repository are presented in this section.

**Fig. 7:**
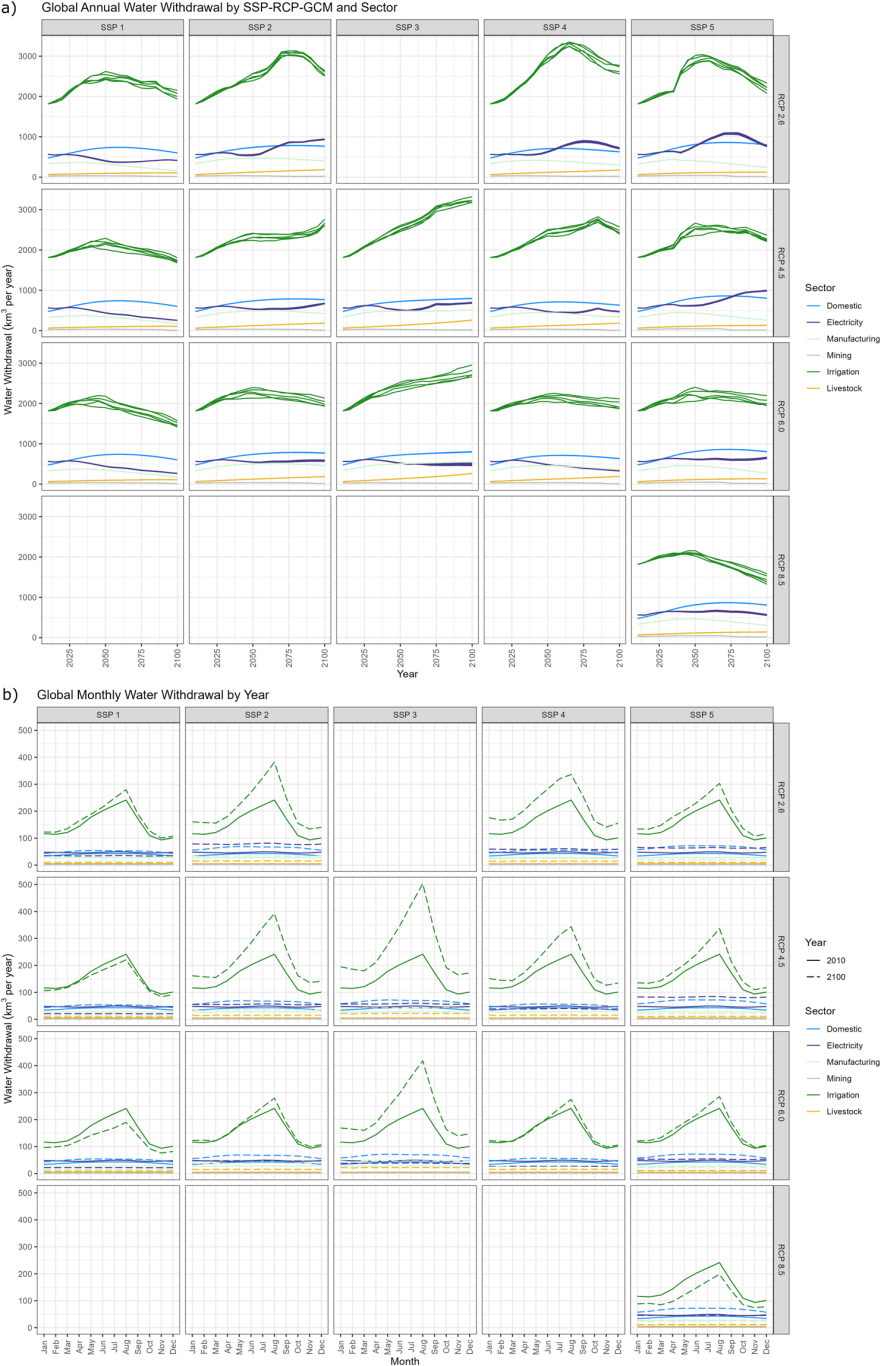
Global water withdrawals for the 75 SSP-RCP-GCM combinations by sector. (**a**) Annual water withdrawals by sector from 2010 to 2100. (**b**) Monthly water withdrawals for 2010 and 2100. Lines of the same color within each plot represent the 5 different GCMs considered.

Figure 7a shows the total annual water withdrawals by sector for each of the 75 SSP-RCP-GCM combinations from 2010 to 2100. Similar figures are available for consumption as well as by crop. Figure 7b shows the sub-annual temporal distribution across the same set of scenarios for 2010 and for 2100. Patterns such as an increase in summer water withdrawals can be seen in such figures.

**Fig. 8:**
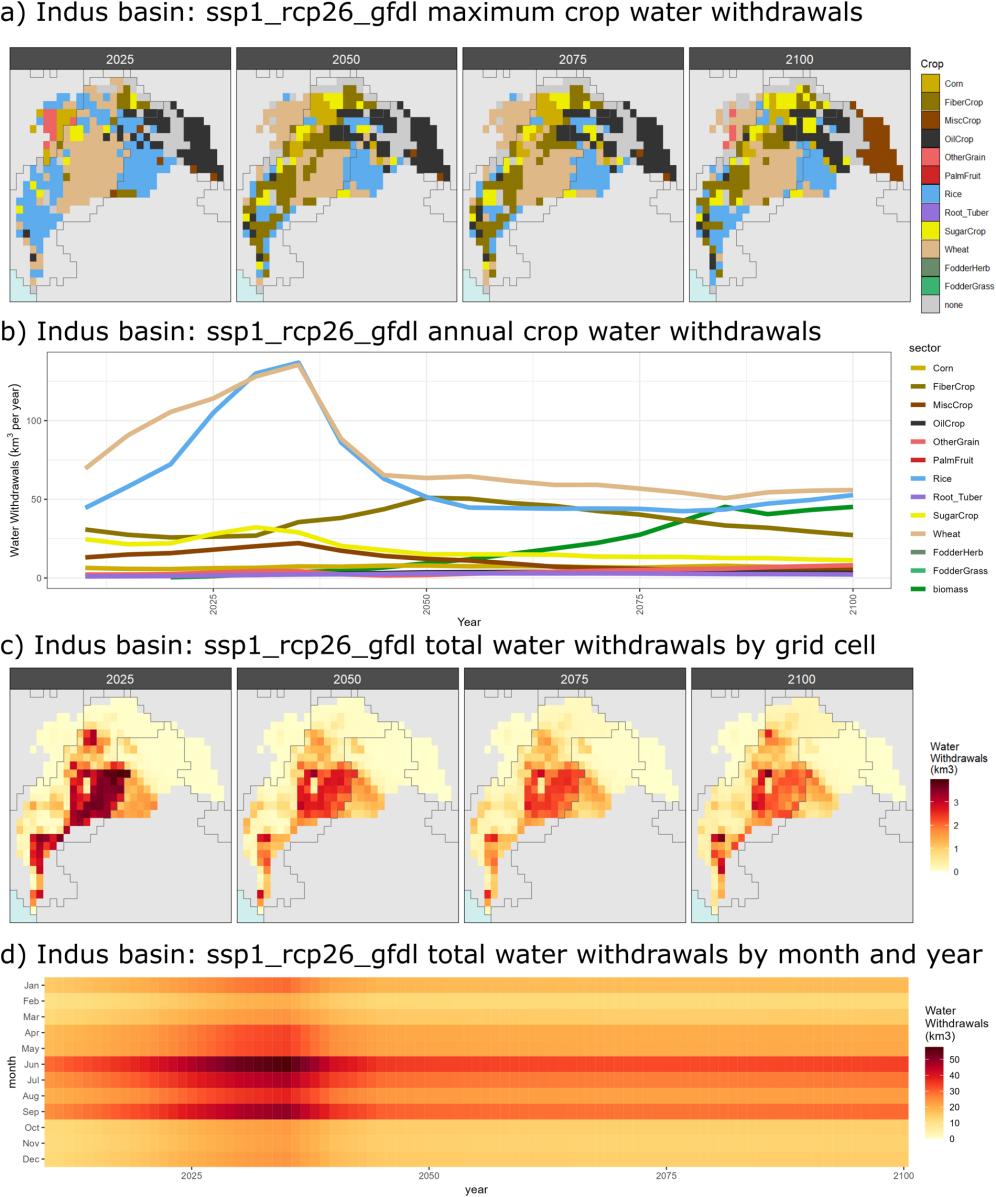
Indus Basin water withdrawals (km^3^) by crop for scenario SSP 1, RCP 2.6, GCM GFDL. (**a**) Showing which crop has the maximum water withdrawals (km^3^) in each grid cell for years 2025, 2050, 2075 and 2100. (**b**) Aggregated water withdrawals (km^3^) by crop in the Indus Basin from 2015 to 2100. (**c**) Showing total water withdrawals (km^3^) in each grid cell for years 2025, 2050, 2075 and 2100. (**d**) Aggregated total water withdrawals (km^3^) in the Indus Basin from 2015 to 2100.

The meta-repository also includes details on three selected basins: the Indus, Nile and Upper Colorado River Basin (U.S.). These are used to show how the data can be used to explore trends and patterns at this finer resolution. Figure 8a,b are examples showing how land-use change impacts which type of crop becomes the dominant water user in the Indus basin over time for the SSP1-RCP2.6-GFDL scenario. Figure 8c,d show the accompanying distribution of total water withdrawals both spatially and temporally. Similar figures are provided in the meta-repository for water consumption as well as for other sectors, crops and scenarios.

We highlight that several developments have been planned in the next release of Tethys to improve the methodologies used to downscale water use for the dataset in this paper. Some of the key planned developments include:

1. Improving the spatial distribution of powerplant water use based on actual and projected powerplant location instead of based on population.
2. Updating the output resolution to 1/8^th^ degrees from the existing ½ degree resolution.
3. Including future population projections to improve on the current methodology which uses a static base year population map even for future years.
4. Improving the downscaling of biomass water use which is currently distributed equally within each region.
5. Making Tethys compatible with GCAM-USA^37^, which allow use of more accurate state-level water use data instead of using national data as inputs to Tethys.
6. Comparing gridded outputs against observational data for individual sectors and regions where data is available.

## Data Availability

Table 3 provides links to all models, data, versions and DOI’s used to generate this dataset.

## References

[1] Mekonnen MM, Hoekstra AY (2016). Four billion people facing severe water scarcity. Science Advances.

[2] UNESCO. *The United Nations World Water Development Report 2022: Groundwater: Making the invisible visible*. https://unesdoc.unesco.org/ark:/48223/pf0000380721 (2022).

[3] Van Vliet MTH (2021). Global water scarcity including surface water quality and expansions of clean water technologies. Environ. Res. Lett..

[4] Graham NT (2020). Humans drive future water scarcity changes across all Shared Socioeconomic Pathways. Environ. Res. Lett..

[5] Hanasaki N (2013). A global water scarcity assessment under Shared Socio-economic Pathways – Part 1: Water use. Hydrol. Earth Syst. Sci..

[6] Hanasaki N (2013). A global water scarcity assessment under Shared Socio-economic Pathways – Part 2: Water availability and scarcity. Hydrol. Earth Syst. Sci..

[7] Hejazi MI (2014). Integrated assessment of global water scarcity over the 21st century under multiple climate change mitigation policies. Hydrology and Earth System Sciences.

[8] Wada Y, Bierkens MFP (2014). Sustainability of global water use: past reconstruction and future projections. Environ. Res. Lett..

[9] Wada Y, Beek LPHV, Wanders N, Bierkens MFP (2013). Human water consumption intensifies hydrological drought worldwide. Environ. Res. Lett..

[10] Yoshikawa, S. *et al*. *An assessment of global net irrigation water requirements from various water supply sources to sustain irrigation: rivers and reservoirs (1960–2000 and 2050).*10.5194/hess-18-4289-2014 (2014).

[11] Veldkamp, T. I. E. Water scarcity at the global and regional scales: unravelling its dominant drivers in historical and future time periods. (2017).

[12] Wada Y, de Graaf IEM, Van Beek LPH (2016). High-resolution modeling of human and climate impacts on global water resources. Journal of Advances in Modeling Earth Systems.

[13] Huang Z (2018). Reconstruction of global gridded monthly sectoral water withdrawals for 1971–2010 and analysis of their spatiotemporal patterns. Hydrology and Earth System Sciences.

[14] Wada Y, Wisser D, Bierkens MFP (2014). Global modeling of withdrawal, allocation and consumptive use of surface water and groundwater resources. Earth System Dynamics.

[15] Mekonnen, M. M. & Hoekstra, A. Y. *Total monthly blue water footprints of production at a 30 × 30 arc minute grid resolution (1996–2005)*. https://waterfootprint.org/en/resources/waterstat/monthly-gridded-blue-water-footprint-statistics/ (2011).

[16] Hofste, R. W. *et al*. Aqueduct 3.0: Updated decision-relevant global water risk indicators. *World Resources Institute: Washington, DC, USA* (2019).

[17] Wild TB (2021). The Implications of Global Change for the Co-Evolution of Argentina’s Integrated Energy-Water-Land Systems. Earth’s Future.

[18] Reed PM (2022). Multisector Dynamics: Advancing the Science of Complex Adaptive Human-Earth Systems. Earth’s Future.

[19] Khan Z, Wild TB, Iyer G, Hejazi M, Vernon CR (2021). The future evolution of energy-water-agriculture interconnectivity across the US. Environ. Res. Lett..

[20] Khan Z (2022). Harvard Dataverse.

[21] Li, X. *et al*. Tethys – A Python Package for Spatial and Temporal Downscaling of Global Water Withdrawals. *Journal of Open Research Software* **6** (2018).

[22] Vernon CR (2018). Demeter – A Land Use and Land Cover Change Disaggregation Model. Journal of Open Research Software.

[23] Van Vuuren DP (2011). The representative concentration pathways: an overview. Climatic Change.

[24] O’Neill BC (2017). The roads ahead: Narratives for shared socioeconomic pathways describing world futures in the 21st century. Global Environmental Change.

[25] ISIMIP. Inter Sectoral Impact Model Intercomparison (ISIMIP) - Input Data and Bias Correction. (2019).

[26] Graham NT (2018). Water Sector Assumptions for the Shared Socioeconomic Pathways in an Integrated Modeling Framework. Water Resources Research.

[27] Chen M (2020). Global land use for 2015–2100 at 0.05° resolution under diverse socioeconomic and climate scenarios. Sci Data.

[28] Narayan K, Di Vittorio A, Vernon C (2021). Zenodo.

[29] Di Vittorio, A., Vernon, C. R. & Shu, S. Moirai Version 3: A Data Processing System to Generate Recent Historical Land Inputs for Global Modeling Applications at Various Scales.

[30] Khan Z (2022). Zenodo.

[31] (2018). Palisades, NY: NASA Socioeconomic Data and Applications Center (SEDAC).

[32] Wint, W. & Robinson, T. *Gridded livestock of the world 2007*. (FAO, Roma (Italia), 2007). 20422554

[33] Wada, Y. *et al*. Global monthly water stress: 2. Water demand and severity of water stress. *Water Resources Research* **47** (2011).

[34] Weedon GP (2014). The WFDEI meteorological forcing data set: WATCH Forcing Data methodology applied to ERA-Interim reanalysis data. Water Resources Research.

[35] Voisin N (2013). One-way coupling of an integrated assessment model and a water resources model: evaluation and implications of future changes over the US Midwest. Hydrology and Earth System Sciences.

[36] Warszawski L (2014). The Inter-Sectoral Impact Model Intercomparison Project (ISI–MIP): Project framework. PNAS.

[37] Binsted M (2022). GCAM-USA v5.3_water_dispatch: Integrated modeling of subnational US energy, water, and land systems within a global framework. Geoscientific Model Development.

[38] World Resources Institute (WRI). WRI Aqueduct. (2021).

[39] Mekonnen, M. & Hoekstra, A. National water footprint accounts: The green, blue and grey water footprint of production and consumption. Volume 1: Main Report. *Daugherty Water for Food Global Institute: Faculty Publications* (2011).

[40] Mekonnen, M. M. & Hoekstra, A. Y. The green, blue and grey water footprint of crops and derived crops products. (2010).

[41] Mekonnen MM, Hoekstra AY (2012). A Global Assessment of the Water Footprint of Farm Animal Products. Ecosystems.

[42] Graham NT (2020). Harvard Dataverse.

[43] Chen M (2020). Zenodo.

[44] Chen, M. & Vernon, C. R. GCAM-Demeter land use dataset at 0.05-degree resolution. *PNNL Datahub* https://data.pnnl.gov/group/nodes/dataset/13192 (2020).

[45] Vernon CR, Chen M (2020). Zenodo.

