# Publisher Correction: Global monthly sectoral water use for 2010–2100 at 0.5° resolution across alternative futures

**DOI:** 10.1038/s41597-023-02168-1

**Published:** 2023-04-28

**Authors:** Zarrar Khan, Isaac Thompson, Chris R. Vernon, Neal T. Graham, Thomas B. Wild, Min Chen

**Affiliations:** 1 Joint Global Change Research Institute, Pacific Northwest National Laboratory, 5825 University Research Ct., Suite 3500, College Park, MD 20740 USA; 2 Department of Forest and Wildlife Ecology, College of Agriculture & Life Sciences, University of Wisconsin – Madison, Russell Labs, 1630 Linden Drive, Madison, WI 53706 USA

**Keywords:** Hydrology, Environmental sciences

Correction to: *Scientific Data* 10.1038/s41597-023-02086-2, published online 11 April 2023

The copyright article for this article was incorrect in the original version and is now attributed to Battelle Memorial Institute.

